# Influence of Subjective/Objective Status and Possible Pathways of Young Migrants’ Life Satisfaction and Psychological Distress in China

**DOI:** 10.3389/fpsyg.2021.612317

**Published:** 2021-05-26

**Authors:** Yi-Chen Chiang, Meijie Chu, Yuchen Zhao, Xian Li, An Li, Chun-Yang Lee, Shao-Chieh Hsueh, Shuoxun Zhang

**Affiliations:** ^1^State Key Laboratory of Molecular Vaccinology and Molecular Diagnostics, School of Public Health, Xiamen University, Xiamen, China; ^2^School of International Business, Xiamen University Tan Kah Kee College, Zhangzhou, China; ^3^Institute of Economics, School of Economics and Wang Yanan Institute for Studies in Economics, Xiamen University, Xiamen, China; ^4^Department of Finance at School of Economics and Wang Yanan Institute for Studies in Economics, Xiamen University, Xiamen, China

**Keywords:** subjective/objective status, city belonging, social participation, psychological distress, life satisfaction, young internal migrants

## Abstract

Young migrants have been the major migrant labor force in urban China. But they may be more vulnerable in quality of life and mental health than other groups, due to their personal characteristic and some social/community policies or management measures. It highlights the need to focus on psychological wellbeing and probe driving and reinforcing factors that influence their mental health. This study aimed to investigate the influence of subjective/objective status and possible pathways of young migrants’ life satisfaction and psychological distress. Data on 9838 young migrants in the China Migrants Dynamic Survey were analyzed by LISREL 8.8. A total of 94.03% migrated for jobs or business. Subjective status, including subjective socioeconomic status, social adaptation, and psychological integration, had positive effects on life satisfaction, whereas social adaptation and psychological integration negatively affected psychological distress. Objective status, including objective socioeconomic status and health insurance, had adverse effects on life satisfaction, whereas they positively affected psychological distress. Social participation and city belonging had only significant positive mediating roles on life satisfaction. It is essential to increase social adaptation and decrease integration stress according to younger internal migrants’ practical needs. It is also necessary to enhance community/social resources and activities in the context of developing sustainability in the community to assist in mental health promotion.

## Introduction

Since the 1980s, rural workers have begun to migrate to cities on a great scale, driven by rapid economic development, socioeconomic transitions, and economic and market policies. Young rural residents move to cities accompanied by an improvement in occupational status, and they tend to migrate further away from home to seek better and more economic opportunities. Young migrants, also called new-generation migrants, born in 1980 or after, have been the major migrant labor force in urban China. Most of them have higher education degrees and more career opportunities than older internal migrants. They prefer to migrate to and have a desire to settle in middle and large cities. However, their income is lower and life expense is higher. The Hukou system is a mandatory household registration system in China that requires each Chinese citizen to register the permanent residence in one place (Tian et al., 2018; Luo et al., 2019). There are two classifications in the Hukou system: hukou type (urban or rural) and hukou locality (whether someone lives in the registered hukou place or not) (Chan, 1994). The educational and employment opportunities, health care, and other government-funded benefits of Chinese are closely related to Hukou, which is difficult to change (Li et al., 2014). Therefore, the Hukou system has become a boundary for the social integration of migrants (Tian et al., 2018). Without local urban Hukou status, the migrants are excluded from several health-related resources and social services, such as the barrier of housing, settlement, and minimum living allowance. In addition, some policies have proposed to provide free medical examinations for the elderly regardless of household registration status as a primary public health service (Zheng et al., 2020). There is rarely a focus on the younger generations of migrants. These circumstances may make the young migrants vulnerable in quality of life and health, and they may experience more stress, social exclusion and social discrimination. Young migrants are more likely to suffer from mental problems, such as anxiety disorders, depression, psychological distress and low self-esteem and subjective wellbeing (Leavey et al., 2004; Wong et al., 2008; Dai et al., 2015; Ma et al., 2020). Moreover, high level of psychological distress was associated with reports of suicide ideation and attempts (Eskin et al., 2016); a low level of life satisfaction has a long-term effect on the risk of suicide (Koivumaa-Honkanen et al., 2001). Young people have a high suicide rate, and suicide was the second-leading cause of death among 15- to 29-year-old people globally in 2016 (World Health Organization (WHO), 2016). Young migrants report a higher prevalence of suicide attempts than non-migrants (McMahon et al., 2017). Migrants’ significant influence on social development and stability highlights the need to focus on mental health status and probe driving and reinforcing factors that influence the mental health of young internal migrants.

Life satisfaction which is one type of subjective wellbeing (Steptoe et al., 2015), is an important indicator for measuring people’ quality of life and related to happiness (Diener et al., 1999; Gamble and Gärling, 2012). Young people have better skills to solve problems, tend to be more resistant to stress and enjoy higher life satisfaction (Lambert et al., 2014; Chui and Wong, 2016). Young adult migrants who move for work-related reasons display improved life satisfaction (Switek, 2016). Do satisfaction in life and work increase due to migration, and how is the mental health of young migrants? Previous studies revealed migrants have low life satisfaction (Hosseini et al., 2017). Life satisfaction not only influence the settlement intention and social participation of migrants but also effects social productivity (Oswald et al., 2015; Huang et al., 2017). Thus, it is necessary to explore influencing factors and develop effective measures to achieve higher life satisfaction. Many scholars have stated that socioeconomic factors (Appleton and Song, 2008; Huang et al., 2017), physical health, participation in politics and welfare (Appleton and Song, 2008), social relationships (Liu et al., 2017), local language proficiency (Beier and Kroneberg, 2013), a sense of integration and social identity all play vital roles in life satisfaction (Huang et al., 2017; Wei and Gao, 2017; Chen et al., 2020b). How do these factors affect life satisfaction? One study revealed that life satisfaction could be affected by community service participation, and identity integration served as a partial mediator in the relationship (Ji et al., 2020). As a manifestation of bad social relationships, social exclusion affects youths’ low life satisfaction by the mediation of resilience and self-esteem (Yıldız and Duy, 2014; Arslan, 2019). However, there is few research investigating the direct and indirect effects of social status, health care, social adaptation and integration on migrants’ life satisfaction.

Psychological distress, which is defined as symptoms of depression and anxiety, is a common psychological issue that requires attention. Extensive research has established that psychological distress is related to many diseases or consequences, such as poor sleep quality (Scott et al., 2014), obesity (Spinosa et al., 2019), emotional depression (Cairney et al., 2007), gastrointestinal symptoms (Clevers et al., 2019), substance use disorders (Lai et al., 2015), posttraumatic stress disorder (PTSD) (Liang et al., 2020), suicidal ideation and suicide attempts (Eskin et al., 2016; Hielscher et al., 2020), and an increased risk of mortality (Russ et al., 2012). Hence, it is crucial to explore and improve the factors that influence depression in migrants, thereby reducing distress. The relationship between socialization factors and psychological distress has been widely analyzed. Socioeconomic status is also associated with psychological distress (Honkaniemi et al., 2020). Lower socioeconomic status (SES) is associated with high psychological distress, and lower subjective social status is related to poor mental health. Poverty and unemployment play an important role in distress (Zechmann and Paul, 2019). Some studies have indicated that traditional SES variables, such as household income, can directly affect psychological distress. Further studies have demonstrated that health care, linguistic familiarity, job demands, job stability, job control, social support and prestige affect one’s psychological distress (Elovainio et al., 2015; Ota et al., 2020; Yang, 2020). A UK study found young migrants had more significant psychological distress, including emotional symptoms and peer problems than young non-migrants (Leavey et al., 2004). Psychological distress among young migrants is worth discussing. Studies examining psychological distress and its influential factors among young migrants in China are relatively scarce.

Social participation can be defined as participation and involvement in social or community activities that provide interactions with others in the community and a platform for fulfilling an individual’s needs for the social integration necessary for wellbeing (Levasseur et al., 2010; Dai et al., 2013; Achdut and Sarid, 2020). Social participation is beneficial for mental health (Sun and Lyu, 2020). Some previous studies demonstrated that social participation could increase life satisfaction (Yuan, 2016; Bai et al., 2017; Au et al., 2020), and reduce psychological distress symptoms (Croezen et al., 2015; Liu et al., 2019). One study found that social participation could explain approximately 11% of psychological health disparity among rural-urban migrants (Sun and Lyu, 2020). Previous studies revealed that social participation mediates the relationship between individual resources and mental health (Dai et al., 2013; Achdut and Sarid, 2020; Sun and Lyu, 2020). However, much evidence for the level of social participation and its association with mental health are in older adults (Kim, 2019; Wang et al., 2020). Few studies have focused on the social participation of young migrants, specifically, young Chinese migrants. But young migrants may have a lower rate of social participation and worse mental health, because of the disparity in community services, social welfare, education and work opportunities, and the structural barriers (Lin et al., 2011). Therefore, it is also important to consider health care services and socioeconomic status when discussing the relationship between social participation and mental health. Additionally, migrants’ city belonging has become an important issue due to their living and working conditions (Zhang et al., 2009). A sense of belonging to a place regarding their hometown and the host city is essential to migrants’ life satisfaction (Chen et al., 2020b). A sense of city belonging had a prominent on psychological distress among first-generation young adult migrants in Australia (Straiton et al., 2019). Moreover, the relationship between social integration and life satisfaction is significantly mediated by city belonging in general migrants (Chen et al., 2020b). However, due to the characteristics of the young migrants in China, whether city belonging mediates other individual and social factors on mental health among young migrants still needs to be addressed.

The stress process model proposed that the stressors produced in the migration process and individual stress vulnerability would affect the mental health of migrants (Pearlin, 1989; de Almeida Vieira Monteiro and Serra, 2011). These stressors include some objective indicators, such as social status and welfare (Pearlin et al., 1981; Pearlin, 1999; Lantz et al., 2005; Min et al., 2005). Besides, some migrants also face the challenge of social integration, such as social exclusion, discrimination experience, and the stressors of social adaptation (Deng and Law, 2020). One of our aims was: to examine the relationship between stressors and mental health among young migrants. So, we proposed two hypotheses: H1) Subjective status, including subjective socioeconomic status, social adaptation, and psychological integration, positively affects life satisfaction and negatively affects psychological distress, while social exclusion is negatively associated with life satisfaction and positively associated with psychological distress; H2) For objective status, objective socioeconomic status and health insurance are positively associated with life satisfaction and negatively affect psychological distress.

According to the self-determination theory, the psychosocial need for relatedness reflects a sense of belonging to the environment and establishes close and meaningful relationships with other people (Deng and Law, 2020). The satisfied psychosocial needs have an important role in protecting or enhancing mental health (Sapmaz et al., 2012). The satisfied needs can increase the individual’s level of life satisfaction, while those unsatisfied needs may cause psychological distress (Ryan et al., 1996; Sheldon and Bettencourt, 2002). According to the stress process model, stress vulnerability can be affected by social resources (Pearlin et al., 1981; Beiser and Hyman, 1997). As a community social resource, social capital includes social participation and city belonging (Lin, 1999; Uphoff et al., 2013). The second purpose of this study is to explore whether social participation and city belonging can serve as buffers between stressors produced in the migration process and mental health, and then improve life satisfaction and reduce psychological distress among young migrants. Based on this, we proposed another two hypotheses: H3 and H4) There are potential mediating roles of social participation and city belonging in the association between other factors and mental health.

This study is novel in that we used a structural equation model (SEM) to measure the complex influencing factors of mental health (life satisfaction and psychological distress) using multiple subjective/objective indicators through path analyses. The SEM enabled us to further examine the influencing factors of social participation and sense of city belonging and their associations with mental health through the structural model.

## Materials and Methods

### Participants

The data were derived from the 2014 China Migrants Dynamic Survey (CMDS), which was conducted by the National Health and Wellness Council of China. The research sample was composed of internal migrants with non-local (county, city) Hukou living in the inflow area for over one month. The survey has adopted a multistage stratified probability proportional to size (PPS) sampling method. The data access and detailed documents can be found at https://chinaldrk.org.cn/wjw/#/achievement/publication/310b8056-6bf2-4243-9062-c60adcab82ee. The available data for this study were derived from a special survey of CMDS in 2014, so-called “social integration and mental health” of migrants. It was designed according to the latest social concern and only conducted in eight cities. According to the comprehensive business index proposed by China Business News Weekly, the eight cities were divided into 3 groups: (1) first-tier city: the Chaoyang District of Beijing and Shenzhen, (2) new first-tier city: Xiamen, Qingdao and Chengdu, and (3) non- first-tier city: Jiaxing, Zhengzhou and Zhongshan. This above-mentioned business index covers total GDP, per capita income, number of colleges and universities, number of Fortune 500 companies, etc., and reflects the city’s overall competitiveness and production technology levels (Sina, 2013; CBNweekly, 2016). This study focused on young migrants born in 1980 and after, and 9838 participants were included in the analysis.

### Measures

#### Economic Status

**Subjective socioeconomic status (S_SES)**, which means one’s perception of their socioeconomic position or rank within a society (Tan et al., 2020), was assessed by the following question: “Compared with people in the whole society, where is your social status?” The scale was depicted as a ladder (rungs 1 to 10), and individual participants were asked to place an “X” on the rung of the ladder that they felt best reflected their social status.

The **objective socioeconomic status (O_SES)** of the participants, which refers to one’s status according to the absolute level of material resources that one possesses (Howell and Howell, 2008), was measured by their monthly household income and level of education. Considering the analysis using the structural equation model (SEM), monthly household income was divided into 3 levels (< 3500 CNY, 3501 CNY - 7000 CNY, and > 7000 CNY). Education level was also categorized into three groups: (1) junior high school or below, (2) high school, and (3) college degree or above.

#### Health Insurance (O_HI)

Health-related resources included social and medical insurance. The study used the following questions: (1) “Which of the following social insurance programs do you use?” and (2) “Do you currently have the following medical insurance?” The values ranged from 1 to 3 (1 = yes; 2 = no; and 3 = not clear). If respondents reported that they had insurance coverage, they were considered underinsured.

#### Social Adaptation (S_SA)

The study included two indicators of social adaptation: dialect familiarity and community harmony. To measure the level of dialect familiarity, the study used the following question: “How well do you know the local dialect?” The response was defined as one of four levels (0 = Not understanding, 1 = Understanding but not speaking, 2 = Understanding and speaking some, and 3 = Understanding and speaking). To measure the level of community harmony, participants were asked the question: “Do you think you or your family get along well with locals?” The response was divided into four levels (0 = Disharmonious, 1 = Generally harmonious, 2 = Relatively harmonious, and 3 = Very harmonious).

#### Psychological Integration (S_PI) and Social Exclusion (S_SE)

Psychological integration was measured in this study using five items, including “I am willing to live with the locals in one block (community).” To measure social exclusion, a three-item scale was used. The items included “I feel the locals don’t like me.” Participants were asked whether certain statements described their situations, and the values ranged from 1 (strongly disagree) to 4 (strongly agree). Cronbach’s alpha values for psychological integration and social exclusion in the present study were both 0.91.

#### City Belonging (CB)

To measure the sense of city belonging in this study, we used five items, including “I feel I belong to this city.” Participants were asked to indicate the extent to whether certain statements described their situations on a four-point Likert scale with responses ranging from 1 (strongly disagree) to 4 (strongly agree). Cronbach’s alpha for city belonging in this study was 0.89.

#### Social Participation (SP)

Social participation in this study was assessed by membership in social organizations and community involvement (Guillen et al., 2011; Fiorillo et al., 2020). Organization member status was measured by the following question: “Are you currently a member of the following organizations locally?” The level of community involvement was assessed by the question “Which of the following activities did you attend locally in 2013 (If immigration to current city short while ago, ask about the situation of this year)?” The response options were “Yes” or “No.” To conduct the SEM, three levels of organization participation and community involvement were defined: 0 = none, 1 = one, and 2 = two or more.

#### Mental Health

We assessed the participants’ mental health based on life satisfaction (LS) and psychological destress (PD). The level of life satisfaction was assessed by the Satisfaction with Life Scale (SWLS), which is based on five items (Diener et al., 1985). The SWLS has demonstrated good psychometric properties in Chinese culture (Ye, 2007). The Chinese version of the SWLS has been shown to have high internal consistency reliability and validity, with Cronbach’s alpha of 0.90 and split-half reliability of 0.70 (Wang et al., 2009, 2017; Ma and Chan, 2015). Psychological distress was assessed using the 6-item Kessler Psychological Distress Scale (K6) (Kessler et al., 2003). The Chinese version of K6 also has been proved to have high reliability and validity in Hong Kong (α = 0.89) (Lee et al., 2012). The values ranged from 1 to 5 (1 = all the time; 2 = most of the time; 3 = sometimes; 4 = occasionally; and 5 = none). Cronbach’s alpha values for SWLS and K6 in the present study were 0.86 and 0.83, respectively.

### Data Analysis

**FIGURE 1:**
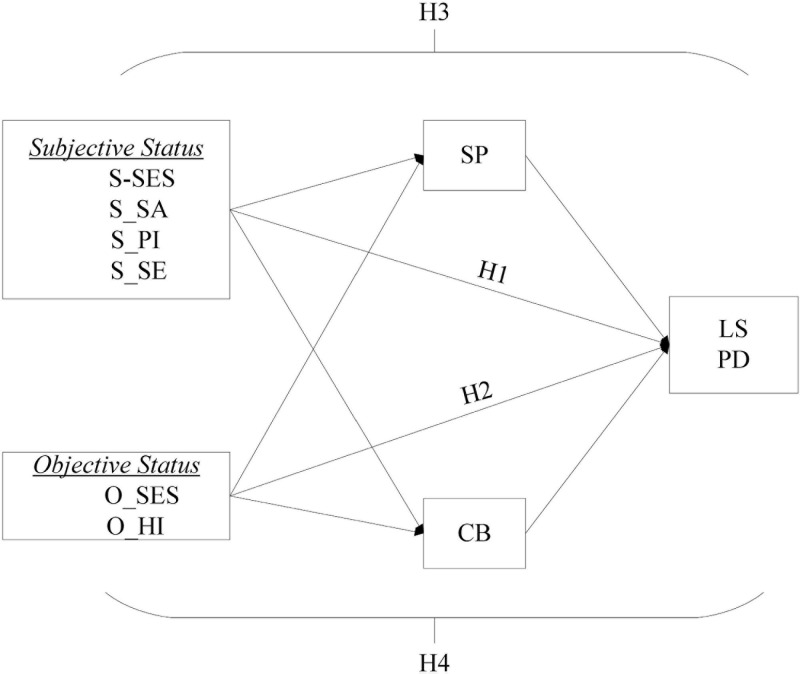
The Hypothesized Path Analysis Model of Life Satisfaction and Psychological Distress in the Sample of Young Internal Migrants. HI to H4 are displayed in the last paragraph of the introduction part. S_SES, Subjective Socioeconomic Status; S_SA, Social Adaptation; S_PL Psychological Integration; S_SE, Social Exclusion; 0_SES, Objective Socioeconomic Status; 0_HI, Health Insurance; SP, Social Participation; CB, City Belonging; LS, Life Satisfaction; PD, Psychological Distress.

The distributions of background characteristics and independent, mediating and dependent variables are expressed in terms of the frequency distribution, mean, maximum and minimum. Structural equation modeling was used to test the mediation effect. The mediating variables were the sense of city belonging and the behavior of social participation. The independent variables were subjective status (including subjective socioeconomic status, social adaptation, psychological integration and social exclusion), and objective status (including objective socioeconomic status and health insurance); and the dependent variable was life satisfaction (as a positive mental health indicator) and psychological distress (as a negative mental health indicator) (Figure 1). The Comparative Fit Index (CFI), Normal Fit Index (NFI) and Incremental Fit Index (IFI) indicate a good fit to the data when values exceed 0.90 (Bentler, 1990; Hu and Bentler, 1999). The Root Mean Squared Error of Approximation (RMSEA) value of < 0.05 indicated a “close fit” (Steiger, 1990; Browne and Cudeck, 1992). In addition, Bollen’s relative fit index (RFI) measures the discrepancy for the model evaluated and for the baseline model, indicating good model-data fit for values close to 1 (Bollen, 1986). A Hoelter’s Critical N (CN) of 200 or better indicated satisfactory model-data fit (Hoelter, 1983). The effects specified were estimated Using the maximum likelihood method. The maximum likelihood method to estimate the polychoric, polyserial, and product–moment correlations were programed in PRELIS2 (Jöreskog and Sörbom, 1996). Statistical analyses were conducted using SAS 9.4 and LISREL 8.8 statistical software. Also, we conducted the correlation matrix of the associations among subjective and objective indicators of the status of young internal Chinese migrants by using LISREL 8.80 (Please refer to Supplementary Table 1). PHI matrix was included in the final SEM estimation. In this study, two methods were used to analyze the discriminant validity among the six latent exogenous variables: (1) the goodness-of-fit of the multi-factor model is significantly better than that of the single-factor model, and (2) the estimated correlation coefficients between any two latent exogenous variables were not more than 0.70 (Please refer to Supplementary Table 1), and the discriminative validity was supported (Kline, 2015).

## Results

### Descriptive Data

Table 1 shows the main demographic characteristics of the participants. Of the 9838 participants in our sample, 94.03% migrated for jobs or business. Most of them were male and were married. The majority of the internal migrants had a junior high school or lower education and had an agricultural Hukou type. Their household income was mainly between 3501 CNY and 7000 CNY. The mean migration duration was 3.12 years. Of the 9838 participants, 27.11% lived in first-tier cities; 34.90% lived in new first-tier cities; 38.00% lived in non-first-tier cities. Table 2 presents the variables’ descriptive statistics in the final structural equation model. The average score for psychological distress was 9.52 (SD = 3.09), and the average score for life satisfaction was 21.41 (SD = 6.20).

**TABLE 1:** Sociodemographic characteristics of the sample (*N* = 9838).

Variables	Category	Frequency	%
Gender			
	Male	5258	53.45
	Female	4580	46.55
City			
	First-tier cities	2667	27.11
	New first-tier cities	3433	34.90
	Non-first-tier cities	3738	38.00
Years of education			
	< High school	4994	50.76
	High school	2892	29.40
	College and > college	1952	19.84
Marital status			
	Married	5835	59.31
	Single, divorced or widow	4003	40.69
Migration range			
	Interprovincial	5320	54.08
	Intercity	4163	42.32
	Intercounty	355	3.61
Hukou type			
	Agricultural	8508	86.48
	Non-agricultural	1213	12.33
	Agricultural to residential	99	1.01
	Non-agricultural to residential	18	0.18
Monthly household income (Mean = 6074.90 CNY)			
	< 3500 CNY	3262	33.16
	3501 ∼ 7000 CNY	4360	44.32
	< 7000 CNY	2216	22.52
Migration duration (Mean = 3.12 years)			
	≤ 1 year	4055	41.22
	2 ∼ 5 years	3971	40.36
	6 ∼10 years	1384	14.07
	> 10 years	428	4.34
Migration reasons			
	Jobs or business	6888	94.03
	Accompanied migration	377	5.15
	Marriage	6	0.08
	Living with relatives	29	0.40
	Birth	7	0.10
	Others	18	0.25

**TABLE 2:** Descriptive data in the final structural equation model.

Variables	Category	Frequency	%
*Objective socioeconomic status*			
Years of education			
	< High school	4994	50.76
	High school	2892	29.40
	College and > college	1952	19.84
Monthly household income			
	< 3500 CNY	3262	33.16
	3501 ∼ 7000 CNY	4360	44.32
	> 7000 CNY	2216	22.52
*Health insurance utilization*			
Social insurance			
	YES	7117	72.34
Medical insurance			
	YES	8563	87.05
*Social participation*			
Organization members			
	None	7049	71.66
	One	1774	18.03
	Two or more	1014	10.31
Activity involvement			
	None	6104	62.05
	One	1794	18.24
	Two or more	1939	19.71
*Social adaptation*			
Dialect familiarity			
	Non-understanding	1503	15.28
	Understanding but not speaking	2196	22.32
	Understanding and speaking some	2189	22.25
	Understanding and speaking	3949	40.14
Community harmony			
	Disharmonious	462	4.70
	Generally harmonious	2524	25.66
	Relatively harmonious	4181	42.50
	Very harmonious	2670	27.14
**Variables**	**Mean**	**SD**	
Subjective socioeconomic status [1 – 10]	4.64	1.66	
Psychological integration [5 – 20]	14.51	2.87	
Social exclusion [3 – 12]	5.59	1.87	
City belonging [5 – 20]	16.17	2.73	
Life satisfaction [5 – 35]	21.41	6.20	
Psychological distress [4 – 20]	6.40	2.17	

*The minimum score and maximum score of every sociopsychological variable are present in brackets.*

### Mediation Analyses

**FIGURE 2:**
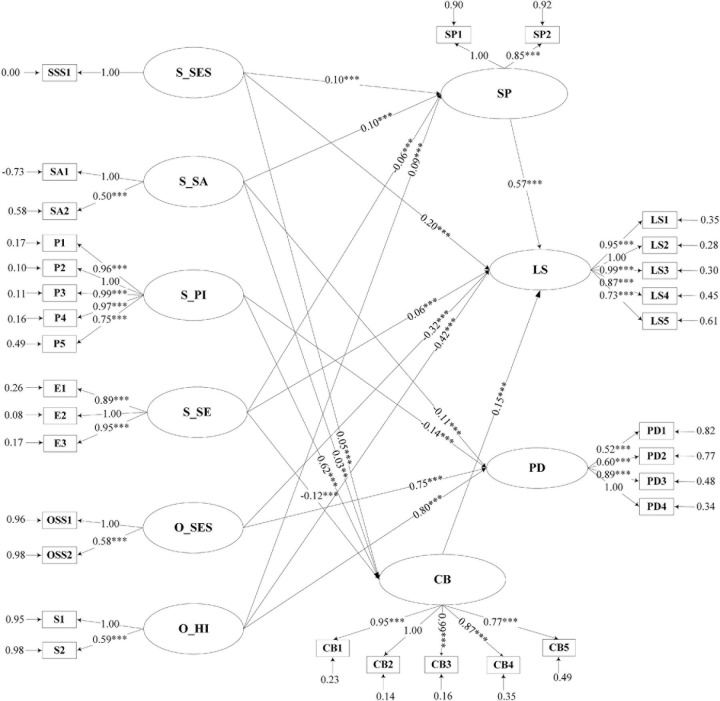
Path Coefficients for the Effects of Subjective/Objective Status on Young Internal Migrants’ Life Satisfaction and Psychological Distress. SSES, Subjective Socioeconomic Status; SSA, Social Adaptation; SPI, Psychological Integration; SSE, Social Exclusion; OSES, Objective Socioeconomic Status; OHI, Health Insurance; SP, Social Participation; CB, City Belonging; LS, Life Satisfaction; PD, Psychological Distress. **p < 0.01, ***p < 0.001.

Based on the hypothesized model, SEM was conducted to analyze the relationships among subjective/objective status, social participant, city belonging, and mental health (life satisfaction and psychological distress). It was found that some of the t-values were less than 1.96 in the initial model. Therefore, the final model was obtained by sequentially deleting O_SES → SP, S_SES → PD, CB → PD, O_SES → CB, O_HI → CB, S_SA → LS, S_PI → LS, SP → PD, the 5^*th*^ indicator of PD, S_PI → SP, the 6^*th*^ indicator of PD and SE → PD. The final model showed improved overall model fit compared to the initial model: RMSEA = 0.035; NFI = 0.99; CFI = 0.99; IFI = 0.99; RFI = 0.98; and CN = 873.23 (Table 3). The final model is shown in Figure 2. Subjective socioeconomic status and social adaptation had a significant positive effect on social participation and city belonging. In contrast, social exclusion had a significant negative effect on these two variables among young migrants. Psychological integration was positively associated with city belonging. Additionally, health insurance was positively associated with social participation. Young migrants with better social participation and city belonging had better life satisfaction (as a positive mental health indicator). Still, there was no positive or negative effect of these variables on psychological distress (as a negative mental health indicator). Specifically, only four out of six questions of the K6 were used to measure psychological distress for young migrants in our final model due to the SEM results. That is, two questions were not significantly associated with psychological distress and dropped out in the final model, one of which was as follows: “About how often during the past 30 days did you feel nervous/hopeless?”

**TABLE 3:** Measures of fit for the life satisfaction and psychological distress model of young internal Chinese migrants.

	Chi-Square	df	RMSEA	NFI	CFI	IFI	RFI	CN
Initial model	7114.10	452	0.039	0.98	0.98	0.98	0.98	726.62
O_SES→ SP	7121.46	453	0.039	0.98	0.98	0.98	0.98	727.36
S_SES → PD	7131.38	454	0.039	0.98	0.98	0.98	0.98	727.84
CB → PD	7139.23	455	0.039	0.98	0.98	0.98	0.98	728.52
O_SES → CB	7150.96	456	0.039	0.98	0.98	0.98	0.98	728.81
O_HI → CB	7160.70	457	0.039	0.98	0.98	0.98	0.98	729.30
S_SA → LS	7162.17	458	0.039	0.98	0.98	0.98	0.98	730.63
S_PI → LS	7165.43	459	0.039	0.98	0.98	0.98	0.98	731.77
SP → PD	7173.77	460	0.039	0.98	0.98	0.98	0.98	732.40
The 5^*th*^ indicator of PD	6300.22	429	0.037	0.98	0.98	0.98	0.98	781.64
S_PI → SP	6309.37	430	0.037	0.98	0.98	0.98	0.98	782.19
The 6^*th*^ indicator of PD	5287.97	400	0.035	0.99	0.99	0.99	0.98	872.77
S_SE → PD ^#^	5297.42	401	0.035	0.99	0.99	0.99	0.98	873.23

*^#^The goodness-of-fit of the Final model. S_SES, Subjective Socioeconomic Status; S_SA, Social Adaptation; S_PI, Psychological Integration; S_SE, Social Exclusion; O_SES, Objective Socioeconomic Status; O_HI, Health Insurance; SP, Social Participation; CB, City Belonging; LS, Life Satisfaction; PD, Psychological Distress.*

First, the pathways from participants’ subjective status to psychological distress and life satisfaction were presented in Table 4. We found a significant positive and direct pathway from subjective socioeconomic status to life satisfaction. We did not find a significant path from subjective socioeconomic status to psychological distress. The direct effect of social adaptation on psychological distress was −0.11 (*p* < 0.001). A negative direct effect of psychological integration on psychological distress was found in the model (β = −0.14, *p* < 0.001). Besides, we found a positive effect of social exclusion on life satisfaction (β = 0.06, *p* < 0.001). Thus, hypothesis 1 (H1) was partially supported.

**TABLE 4:** Direct and indirect effects of socialization factors on life satisfaction and psychological distress in young migrants.

Variable	Life satisfaction			Psychological distress		
	Direct effect	Indirect effect	Total effect	Direct effect	Indirect effect	Total effect
***Subjective status***						
Subjective socioeconomic status	0.20***	0.07***	0.27***	–	–	–
Social adaptation	–	0.06***	0.06***	−0.11***	–	−0.11***
Psychological integration	–	0.09***	0.09***	−0.14***	–	−0.14***
Social exclusion	0.06***	- 0.05***	0.01	–	–	–
***Objective status***						
Objective socioeconomic status	−0.32***	–	−0.32***	0.75***	–	0.75***
Health insurance utilization	−0.42***	0.05**	−0.36***	0.80***	–	0.80***
***Mediation variables***						
Social participation	0.57***	–	0.57***	–	–	–
City belonging	0.15***	–	0.15***	–	–	–

**p < 0.01 (two-tailed significance tests); ***p < 0.001(two-tailed significance tests).

Second, the model indicated that the direct effect of objective socioeconomic status on life satisfaction was negative and that the direct effect of it on psychological distress was positive. Health insurance had a direct effect on mental health: receiving health insurance contributed to a lower level of life satisfaction (β = −0.42, *p* < 0.001) and higher psychological distress (β = 0.80, *p* < 0.001). Thus, hypothesis 2 (H2) was not supported (Table 4).

Finally, some of the Chinese internal migrants’ subjective and objective status not only directly affected their mental health (life satisfaction and psychological distress), but also indirectly affected their life satisfaction through the behavior of social participation and a sense of city belonging. Significant positive indirect effects of subjective socioeconomic status on life satisfaction via social participation and city belonging were identified from the model. The total effect of subjective socioeconomic status on life satisfaction was 0.27 and significant. Besides, we found a positive indirect effect of social adaptation on life satisfaction (β = 0.06, *p* < 0.001), which was mediated by a sense of city belonging and social participation. The level of psychological integration had a positive indirect effect on life satisfaction, the effect was 0.09 and significant (*p* < 0.001). A serial mediation pathway was also identified from the model between social exclusion and life satisfaction via social participation and a sense of city belonging. The indirect effect was −0.05 (*p* < 0.001). However, the total effect of social exclusion on life satisfaction was not significant due to the combination of direct and indirect effects. Migrants’ health insurance had a significant (*p* < 0.001) indirect effect on life satisfaction via social participation. The total effect of health insurance on life satisfaction was −0.36 (*p* < 0.001). To sum, the mediation hypotheses (H3 and H4) were partially supported (Table 4).

## Discussion

This study focused on young internal migrants or so-called new-generation migrants. The main reason for immigration was to work. Their mental health problems may lead to bad behaviors that affect their quality of life and social-economic development. We examined the influence of subjective/objective status of young migrants’ life satisfaction and psychological distress in China, as well as social participation and the sense of city belonging as mediators of these associations by using the structural equation model. Our study mainly has the following theoretical contributions and practical implications for government and community management of migrants:

First, we found some subjective indicators directly affect young migrants’ life satisfaction and psychological distress. Subjective socioeconomic status had a significant positive association with life satisfaction, which is consistent with the findings of previous studies (Appleton and Song, 2008; Huang et al., 2017; You et al., 2019). For young migrants, social interaction may also be a source of stress. Current youth tend to pay more attention to mobile networks than social interaction with others face to face; thus, higher social exclusion may not lead to lower life satisfaction among young migrants. However, young migrants with higher levels of social adaptation and psychological integration have less psychological distress. These relationships were also found in other studies (Huang et al., 2017; Chen et al., 2020b). It indicated that we need to take young migrants’ personal characteristics and current net culture should be incorporated into mental health promotion initiatives.

This study revealed a negative association between objective socioeconomic status and life satisfaction, and a positive effect of objective socioeconomic status on psychological distress. Previous studies on this relationship had mixed results. Some studies demonstrated that individuals with higher education (Hashemi et al., 2020) may have feelings of self-confidence and self-estimation (Cunado and Perez De Gracia, 2012) and have a higher income. Hence, they are happier and have higher life satisfaction. However, one study advocated that education was not found to predict life satisfaction (Hashemi et al., 2020). Our research supported one study which revealed education and household income were negatively associated with self-rated mental health (Honjo et al., 2006). For young migrants, the gap between reality and ideals leads to lower life satisfaction. Higher objective socioeconomic status may have opposite effects on mental health among young migrants due to excessive social pressure (Tesser et al., 1983; Huang et al., 2017) and intense aspirations to settle down (Chen et al., 2016). Our findings suggest that the formulation of socio-economic policy and welfare programs should take subjective socioeconomic status into consideration rather than objective socioeconomic status alone. Future researchers who design similar studies may consider using more indicators of subjective and objective socioeconomic status identifying other antecedent conditions to further discuss the association between socioeconomic status and mental health. Besides, many young migrants are confident about their health status and reluctantly pay for health insurance, or they think it is unnecessary to participate in insurance. Young migrants who are insured may have lower psychological health, and compulsory health insurance may worsen this phenomenon (Huang et al., 2020). In view of the concept that many immigrants with health insurance perceived they had a better health condition (Cloos et al., 2020), we recommend health agencies and stakeholders should focus on improving the health and policy literacy of young migrants to promote understanding of the importance of health insurance.

Second, more social participation and a sense of city belonging can improve life satisfaction, which is consistent with previous studies (Appleton and Song, 2008; Chen et al., 2016, 2020b). Encouraging more young migrants to participate in community activities and local clubs is a good way to improve mental health. Moreover, social participation and a sense of city belonging were included as variables in the theoretical model in this paper to analyze the impact of their mediating effects. The model suggests that subjective status, including subjective socioeconomic status, social adaptation, psychological integration and social exclusion; and objective status including health insurance has indirect associations with life satisfaction.

People with a higher degree of subjective socioeconomic status and health insurance may have more self-esteem and self-confidence and less stress, so they engage more in various forms of social participation or benefit from these interactions and activities, leading to more life satisfaction. As social participation is a key contributor to social inclusion (Filia et al., 2019), migrants with higher social adaptation and less subjective social exclusion are closer to communities and neighborhoods, which plays a crucial role in migrants’ social participation in urban societies. Also, migrants with higher subjective SES are more likely to choose family reunions in cities, leading to a greater sense of city belonging (Quassoli and Dimitriadis, 2019; Chen et al., 2020a). In addition, it is reasonable to expect that migrants with higher levels of social adaptation would be socially and culturally more accepted by urban residents; thus, they are more likely to develop a sense of belonging in the city than other migrants (Wang and Fan, 2012). The mediating roles of city belonging in the association between integration and life satisfaction were consistent with previous studies (Chen et al., 2020b). Young people have less social exclusion and emplace relationships with family and some friends as key to strong belonging, while young migrants who experience social exclusion expressed an ambivalent sense of belonging (Zevallos, 2008). Young migrants should be encouraged to join more social or community organizations and participate in more activities, thereby increasing their opportunities for social participation and improving their sense of city belonging.

Finally, it is worth mentioning that the results of the positive direct effect and negative indirect effect of social exclusion on life satisfaction revealed that improvements in life satisfaction should be informed by young migrants’ characteristics and needs, followed by the development of practical social interaction platforms. In addition, the results showed that subjective and objective status had no indirect association with psychological distress through social participation and a sense of city belonging in our study. Other characteristics including self-esteem, self-confidence, mastery and improvement of skills, may be more important to decrease psychological distress among young migrants. Previous studies revealed that formal or informal social participation and social support themselves were not directly or indirectly associated with psychological distress in youth (Child and Lawton, 2020). Future research should be introduced to improve the model for psychological distress among young internal migrants.

We hope that implications for policymakers and community health management to develop opportunities to increase social adaptation and decrease integration stress in young migrants who prefer to interact and socialize with others could aid in improving their mental health; and enhance community/social resources and activities in the context of developing sustainability in the community, can be drawn from this study. The community manager and organizations can provide the actual job assistance or interaction for the young migrants to increase eudaimonic work wellbeing and life satisfaction. Our findings, along with those from other studies, demonstrate that enhancing of social participation practices and a sense of city belonging among low subjective socioeconomic status individuals, lower social adaptation and integration, and a greater sense of exclusion are recommended. It is vital to create more rights and development opportunities for migrants to enhance one’s sense of city belonging (Bauder, 2016).

This study has several limitations. First, the cross-sectional design of the study limits any causal inferences among the variables of interest. Future research could specify the temporal sequences according to a longitudinal study design. Mediation analysis is generally used to assess longitudinal processes. Therefore, longitudinal research designs would allow us to examine the potential causal effects of subjective and objective status on mental health. Second, mental health outcomes were assessed using self-report scales or questions, resulting in biased data. However, previous studies also used self-reported psychological distress and life satisfaction to measure mental health (South et al., 2018) and wellbeing (Elgar et al., 2015; Viner et al., 2019; Scheim et al., 2020). Future research data should be collected combining subjective and objective methods or technologies. Within the context of these limitations, the results of this study indicated that social participation and city belonging can mediate the association between subjective/objective status characteristics and mental health. More studies should be conducted to highlight further opportunities to improve social participation and city belonging to increase life satisfaction, and reduce psychological distress systems in young migrants. According to our findings, it is necessary to combine government, community management, and other stakeholders to relieve the psychological discomfort of migrants.

## Conclusion

In conclusion, the current study reports the mediating role of social participation and city belonging in the relationship between subjective/objective status and life satisfaction (as a positive mental health indicator), as well as psychological distress (as a negative mental health indicator) among young migrants in developing countries. The results of this study emphasize the importance of developing opportunities to activate participation of young migrants in social or community activities, for example, providing various opportunities for social network with the help of new media can help to increase the migrant workers’ integration and social adaptation to improve their mental health. It is also necessary to properly enhance community/social resources in the context of developing sustainability in the community.

## Disclosure

The views expressed in this study were those of the authors and not necessarily those of the National Health and Wellness Council of China.

## Data Availability Statement

The data analyzed in this study is subject to the following licenses/restrictions: The data that support the findings of this study are available from the National Health and Wellness Council of China. We had to sign a legally binding agreement with the Commission that we will not share any original data with any third parties. Requests to access these datasets should be directed to http://www.moh.gov.cn/ldrks/s7846r/201410/ee63c32ca4b7443faf2feeb14ce88874.shtml.

## Ethics Statement

Our de-identified data derived from the China Migrants Dynamic Survey which was approved by the National Health and Family Planning Commission Ethics Review Board. All participants provided informed consent. As this involved analyzing de-identified existing data, this study did not receive ethical committee approval.

## Author Contributions

SZ and Y-CC had full access to all of the data in the study and take responsibility for the integrity of the data and the accuracy of the data analysis. SZ responsible for ensuring that the descriptions are accurate and agreed by all authors. Y-CC, MC, C-YL, S-CH and SZ conceived the manuscript. Y-CC and MC formal analysis. MC and YZ wrote first draft. XL, AL, C-YL, S-CH, and SZ contributed to revisions and rewriting. Y-CC acquisition of funding. All authors approved the final version, and all take responsibility for its content.

## Conflict of Interest

The authors declare that the research was conducted in the absence of any commercial or financial relationships that could be construed as a potential conflict of interest.

## References

[B1] Achdut N. Sarid O. (2020). Socio-economic status, self-rated health and mental health: the mediation effect of social participation on early-late midlife and older adults. *Isr. J. Health. Policy. Res* 9:49. 10.1186/s13584-019-0359-8 31992363 PMC6988248

[B2] Appleton S. Song L. (2008). Life satisfaction in urban China: components and determinants. *World. Dev.* 36 2325–2340. 10.1016/j.worlddev.2008.04.009

[B3] Arslan G. (2019). Mediating role of the self-esteem and resilience in the association between social exclusion and life satisfaction among adolescents. *Pers. Individ. Dif.* 151:109514. 10.1016/j.paid.2020.109955

[B4] Au A. Lai D. W. L. Yip H. M. Chan S. Lai S. Chaudhury H. (2020). Sense of community mediating between age-friendly characteristics and life satisfaction of community-dwelling older adults. *Front. Psychol.* 11:10. 10.3389/fpsyg.2020.00086 32194465 PMC7064721

[B5] Bai X. Hung K. Lai D. W. L. (2017). The role of travel in enhancing life satisfaction among Chinese older adults in Hong Kong. *Ageing Soc.* 37 1824–1848. 10.1017/S0144686X16000611

[B6] Bauder H. (2016). Possibilities of Urban Belonging. *Antipode* 48 252–271. 10.1111/anti.12174

[B7] Beier H. Kroneberg C. (2013). Language boundaries and the subjective well-being of immigrants in Europe. *J. Ethn. Migr. Stud.* 39 1535–1553. 10.1080/1369183X.2013.833685

[B8] Beiser M. Hyman I. (1997). Refugees’ time perspective and mental health. *Am. J. Psychiatry* 154 996–1002. 10.1176/ajp.154.7.996 9210752

[B9] Bentler P. M. (1990). Comparative fit indexes in structural models. *Psychol. Bull.* 107 238–246. 10.1037/0033-2909.107.2.238 2320703

[B10] Bollen K. (1986). Sample size and bentler and Bonett’s nonnormed fit index. *Psychometrika* 51 375–377. 10.1007/BF02294061

[B11] Browne M. W. Cudeck R. (1992). Alternative ways of assessing model fit. *Soc. Methods Res.* 21 230–258. 10.1177/004912419021002005

[B12] Cairney J. Veldhuizen S. Wade T. J. Kurdyak P. Streiner D. L. (2007). Evaluation of 2 measures of psychological distress as screeners for depression in the general population. *Can. J. Psychiatry.* 52 111–120. 10.1177/070674370705200209 17375867

[B13] CBNweekly (2016). *China City Business Charm Ranking: Reclassification of Chinese Cities (2016).* Available online at: https://www.cbnweek.com/articles/normal/14189 (accessed March 4, 2021). 10.1177/070674370705200209 17375867

[B14] Chan K. W. (1994). *Cities with Invisible Walls: Reinterpreting Urbanization in Post-1949 China.* New York, NY: Oxford University Press.

[B15] Chen H. Wang X. Liu Y. Liu Y. Chen H. S. Wang X. P. (2020a). Migrants’ choice of household split or reunion in China’s urbanisation process: The effect of objective and subjective socioeconomic status. *Cities* 102:102669. 10.1016/j.cities.2020.102669

[B16] Chen H. Zhu Z. Chang J. Gao Y. Chen H. S. Zhu Z. J. (2020b). The effects of social integration and hometown identity on the life satisfaction of Chinese rural migrants: the mediating and moderating effects of a sense of belonging in the host city. *Health Qual. Life Outcomes* 18:171. 10.1186/s12955-020-01415-y 32505205 PMC7275305

[B17] Chen H. Zhu Z. Sun D. Wang X. (2016). The physical and psychological health of migrants in Guangzhou, China: how does neighborhood matter? *Inquiry* 53:0046958016668065. 10.1177/0046958016668065 27637270 PMC5798746

[B18] Child S. T. Lawton L. E. (2020). Personal networks and associations with psychological distress among young and older adults. *Soc. Sci. Med.* 246:112714. 10.1016/j.socscimed.2019.112714 31864967 PMC7025742

[B19] Chui W. Wong M. Y. H. (2016). Gender differences in happiness and life satisfaction among adolescents in Hong Kong: relationships and self-concept. *Soc. Indic. Res.* 125 1035–1051. 10.1007/s11205-015-0867-z

[B20] Clevers E. Törnblom H. Simrén M. Tack J. Van Oudenhove L. (2019). Relations between food intake, psychological distress, and gastrointestinal symptoms: a diary study. *U.Eur. Gastroenterol. J.* 7 965–973. 10.1177/2050640619839859 31428421 PMC6683644

[B21] Cloos P. Ndao E. M. Aho J. Benoit M. Fillol A. Munoz-Bertrand M. (2020). The negative self-perceived health of migrants with precarious status in Montreal, Canada: a cross-sectional study. *PLoS One* 15:e0231327. 10.1371/journal.pone.0231327 32271827 PMC7145148

[B22] Croezen S. Avendano M. Burdorf A. van Lenthe F. J. (2015). Social participation and depression in old age: a fixed-effects analysis in 10 european countries. *Am. J. Epidemiol.* 182 168–176. 10.1093/aje/kwv015 26025236 PMC4493978

[B23] Cunado J. Perez De Gracia F. (2012). Does education affect happiness? *Evid. Spain. Soc. Indic. Res.* 108 1–12. 10.1007/s11205-011-9874-x

[B24] Dai B. Zhang B. Li J. (2013). Protective factors for subjective well-being in chinese older adults: the roles of resources and activity. *J. Happiness Stud.* 14 1225–1239. 10.1007/s10902-012-9378-7

[B25] Dai J. Zhong B. L. Xiang Y. T. Chiu H. F. Chan S. S. Yu X. (2015). Internal migration, mental health, and suicidal behaviors in young rural Chinese. *Soc. Psychiatry Psychiatr. Epidemiol.* 50 621–631. 10.1007/s00127-014-0985-y 25403568 PMC4536925

[B26] de Almeida Vieira Monteiro A. P. Serra A. V. (2011). Vulnerability to stress in migratory contexts: a study with Eastern European immigrants residing in Portugal. *J. Immigr. Minor. Health* 13 690–696. 10.1007/s10903-011-9451-z 21287274

[B27] Deng Z. Law Y. W. (2020). Rural-to-urban migration, discrimination experience, and health in China: evidence from propensity score analysis. *PLoS One* 15:e0244441. 10.1371/journal.pone.0244441 33370369 PMC7769422

[B28] Diener E. Emmons R. A. Larsen R. J. Griffin S. (1985). The satisfaction with life scale. *J. Pers. Assess.* 49 71–75. 10.1207/s15327752jpa4901_13 16367493

[B29] Diener E. Suh E. Lucas R. Smith H. (1999). Subjective well-being: three decades of progress. *Psychol. Bull.* 125 276–302. 10.1037/0033-2909.125.2.276

[B30] Elgar F. J. Pförtner T.-K. Moor I. De Clercq B. Stevens G. W. J. M. Currie C. (2015). Socioeconomic inequalities in adolescent health 2002–2010: a time-series analysis of 34 countries participating in the Health Behaviour in School-aged Children study. *The Lancet* 385 2088–2095. 10.1016/S0140-6736(14)61460-4 25659283

[B31] Elovainio M. Heponiemi T. Jokela M. Hakulinen C. Presseau J. Aalto A. M. (2015). Stressful work environment and wellbeing: what comes first? *J. Occup. Health Psychol.* 20 289–300. 10.1037/a0038684 25705911

[B32] Eskin M. Sun J. M. Abuidhail J. Yoshimasu K. Kujan O. Janghorbani M. (2016). Suicidal behavior and psychological distress in university students: a 12-nation study. *Arch. Suicide Res.* 20 369–388. 10.1080/13811118.2015.1054055 26954847

[B33] Filia K. Jackson H. Cotton S. Killackey E. (2019). Understanding what it means to be socially included for people with a lived experience of mental illness. *Int. J. Soc. Psychiatr.* 65 413–424. 10.1177/0020764019852657 31159628

[B34] Fiorillo D. Lavadera G. L. Nappo N. (2020). Individual heterogeneity in the association between social participation and self-rated health: a panel study on BHPS. *Soc. Indic. Res.* 151 645–667. 10.1007/s11205-020-02395-8 32836671 PMC7266383

[B35] Gamble A. Gärling T. (2012). The relationships between life satisfaction, happiness, and current mood. *J. Happinesss Stud.* 13 31–45. 10.1007/s10902-011-9248-8

[B36] Guillen L. Coromina L. Saris W. E. (2011). Measurement of social participation and its place in social capital theory. *Soc. Indic. Res.* 100 331–350. 10.1007/s11205-010-9631-6

[B37] Hashemi N. Marzban M. Sebar B. Harris N. (2020). Perceived discrimination and subjective well-being among Middle Eastern migrants in Australia: the moderating role of perceived social support. *Int. J. Soc. Psychiatr.* 20764020940740. 10.1177/0020764020940740 32635789

[B38] Hielscher E. DeVylder J. Hasking P. Connell M. Martin G. Scott J. G. (2020). Mediators of the association between psychotic experiences and future non-suicidal self-injury and suicide attempts: results from a three-wave, prospective adolescent cohort study. *Eur. Child Adolesc. Psychiatry* 10.1007/s00787-020-01593-6 [Epub ahead of print]. 32712716

[B39] Hoelter J. W. (1983). The analysis of covariance-structures - goodness-of-fit indexes. *Soc. Methods Res.* 11 325–344. 10.1177/0049124183011003003

[B40] Honjo K. Kawakami N. Takeshima T. Tachimori H. Ono Y. Uda H. (2006). Social class inequalities in self-rated health and their gender and age group differences in Japan. *J. Epidemiol.* 16 223–232. 10.2188/jea.16.223 17085872 PMC7683699

[B41] Honkaniemi H. Juarez S. P. Katikireddi S. V. Rostila M. (2020). Psychological distress by age at migration and duration of residence in Sweden. *Soc. Sci. Med.* 250:112869. 10.1016/j.socscimed.2020.112869 32120203 PMC8325349

[B42] Hosseini A. Kakuma R. Ghazinour M. Davern M. Evans W. Minas H. (2017). Migration experience, resilience and depression: a study of Iranian immigrants living in Australia. *Int. J. Cult. Ment. Health* 10 108–120. 10.1080/17542863.2016.1270977 [Epub ahead of print].

[B43] Howell R. T. Howell C. J. (2008). The relation of economic status to subjective well-being in developing countries: a meta-analysis. *Psychol. Bull.* 134 536–560. 10.1037/0033-2909.134.4.536 18605819

[B44] Hu L. T. Bentler P. M. (1999). Cutoff criteria for fit indexes in covariance structure analysis: conventional criteria versus new alternatives. *Struct. Equal. Model.* 6 1–55. 10.1080/10705519909540118

[B45] Huang J. L. Yuan L. Liang H. (2020). Which matters for medical utilization equity under universal coverage: insurance system, region or SES. *Int. J. Environ. Res. Public Health.* 17:4131. 10.3390/ijerph17114131 32531889 PMC7312584

[B46] Huang S. Hou J. Sun L. Dou D. Liu X. Zhang H. (2017). The effects of objective and subjective socioeconomic status on subjective well-being among rural-to-urban migrants in china: the moderating role of subjective social mobility. *Front. Psychol.* 8:819. 10.3389/fpsyg.2017.00819 28588531 PMC5439243

[B47] Ji X. Ji X. W. Chui C. H. K. Ni S. G. Dong R. (2020). Life satisfaction of rural migrant workers in Urban China: the roles of community service participation and identity integration. *J. Soc. Serv. Res.* 46 273–282. 10.1080/01488376.2018.1555110

[B48] Jöreskog K. Sörbom D. (1996). *PRELIS 2: User’s Reference Guide*, 3rd Edn. Chicago, IL: Scientific Software International.

[B49] Kessler R. C. Barker P. R. Colpe L. J. Epstein J. F. Gfroerer J. C. Hiripi E. (2003). Screening for serious mental illness in the general population. *Arch. Gen. Psychiatry* 60 184–189. 10.1001/archpsyc.60.2.184 12578436

[B50] Kim S. (2019). A study of married immigrant women’s experience of community participation based on interculturalism – focused on social economic community. *Res. Inst. Life Cult. Sogang Univ.* 52 57–84. 10.17924/solc.2019.52.57

[B51] Kline R. B. (2015). *Principles and Practice of Structural Equation Modeling*, 4th Edn. New York, NY: Guilford Press.

[B52] Koivumaa-Honkanen H. Honkanen R. Viinamäki H. Heikkilä K. Kaprio J. Koskenvuo M. (2001). Life satisfaction and suicide: a 20-year follow-up study. *Am. J. Psychiatry* 158 433–439. 10.1176/appi.ajp.158.3.433 11229985

[B53] Lai H. M. X. Cleary M. Sitharthan T. Hunt G. E. (2015). Prevalence of comorbid substance use, anxiety and mood disorders in epidemiological surveys, 1990–2014: a systematic review and meta-analysis. *Drug Alcohol Depend.* 154 1–13. 10.1016/j.drugalcdep.2015.05.031 26072219

[B54] Lambert M. Fleming T. Ameratunga S. Robinson E. Crengle S. Sheridan J. (2014). Looking on the bright side: an assessment of factors associated with adolescents’ happiness. *Adv. Ment. Health* 12 101–109. 10.1080/18374905.2014.11081888

[B55] Lantz P. M. House J. S. Mero R. P. Williams D. R. (2005). Stress, life events, and socioeconomic disparities in health: results from the Americans’ changing lives study. *J. Health Soc. Behav.* 46 274–288. 10.1177/002214650504600305 16259149

[B56] Leavey G. Hollins K. King M. Barnes J. Papadopoulos C. Grayson K. (2004). Psychological disorder amongst refugee and migrant schoolchildren in London. *Soc. Psychiatry Psychiatr. Epidemiol.* 39 191–195. 10.1007/s00127-004-0724-x 14999451

[B57] Lee S. Tsang A. Ng K. L. Ma Y. L. Guo W. Mak A. (2012). Performance of the 6-item Kessler scale for measuring serious mental illness in Hong Kong. *Compr. Psychiatry* 53 584–592. 10.1016/j.comppsych.2011.10.001 22104556

[B58] Levasseur M. Richard L. Gauvin L. Raymond E. (2010). Inventory and analysis of definitions of social participation found in the aging literature: Proposed taxonomy of social activities. *Soc. Sci. Med.* 71 2141–2149. 10.1016/j.socscimed.2010.09.041 21044812 PMC3597625

[B59] Li J. Gu Y. F. Zhang C. C. (2014). Hukou-Based Stratification in Urban China’s Segmented Economy. *Chin. Soc. Rev.* 47 154–176. 10.1080/21620555.2014.990326

[B60] Liang L. Gao T. Ren H. Cao R. Qin Z. Hu Y. (2020). Post-traumatic stress disorder and psychological distress in Chinese youths following the COVID-19 emergency. *J. Health Psychol.* 25 1164–1175. 10.1177/1359105320937057 32627606 PMC7342938

[B61] Lin D. H. Li X. M. Wang B. Hong Y. Fang X. Y. Qin X. O. (2011). Discrimination, perceived social inequity, and mental health among rural-to-urban migrants in China. *Commun. Ment. Health J.* 47 171–180. 10.1007/s10597-009-9278-4 20033772 PMC2891847

[B62] Lin N. J. C. (1999). Building a network theory of social capital. *Connections* 22 28–51.

[B63] Liu H. Y. Fang B. Y. Chan J. L. Lou V. W. Q. (2019). Continued social participation protects against depressive symptoms across the retirement transition: longitudinal evidence from three waves of the China Health and Retirement longitudinal Survey. *Geriatr. Gerontol. Int.* 19 972–976. 10.1111/ggi.13752 31397048

[B64] Liu Y. Q. Zhang F. Z. Liu Y. Li Z. G. Wu F. L. (2017). The effect of neighbourhood social ties on migrants’ subjective wellbeing in Chinese cities. *Habitat. Int.* 66 86–94. 10.1016/j.habitatint.2017.05.011

[B65] Luo J. Chen Y. F. He H. R. Gao G. L. (2019). Hukou identity and fairness in the ultimatum game. *Theory Decis.* 87 389–420. 10.1007/s11238-019-09700-z

[B66] Ma C. Qu Z. P. Xu Z. M. (2020). Internal migration and mental health: an examination of the healthy migration phenomenon in China. *Popul. Res. Policy Rev.* 39 493–517. 10.1007/s11113-019-09552-z

[B67] Ma W. W. K. Chan C. K. (2015). Online knowledge sharing and psychological well-being among chinese college students. *J. Commun. Educ.* 2 31–38.

[B68] McMahon E. M. Corcoran P. Keeley H. Cannon M. Carli V. Wasserman C. (2017). Mental health difficulties and suicidal behaviours among young migrants: multicentre study of European adolescents. *BJPsych. Open* 3 291–299. 10.1192/bjpo.bp.117.005322 29234521 PMC5707442

[B69] Min M. O. Townsend A. L. Miller B. Rovine M. J. (2005). Supplemental private health insurance and depressive symptoms in older married couples. *Int. J. Aging Hum. Dev.* 61 293–312. 10.2190/21la-xqce-bkjf-mc17 16320444

[B70] Oswald A. J. Proto E. Sgroi D. (2015). Happiness and productivity. *J. Labor Econ.* 33 789–822.

[B71] Ota M. Takeda S. Pu S. H. Matsumura H. Araki T. Hosoda N. (2020). The relationship between cognitive distortion, depressive symptoms, and social adaptation: a survey in Japan. *J. Affect. Disord* 265 453–459. 10.1016/j.jad.2020.01.09410.1016/j.jad.2020.02.045 32090772

[B72] Pearlin L. I. (1989). The sociological study of stress. *J. Health Soc. Behav.* 30 241–256. 10.2307/2136956 2674272

[B73] Pearlin L. I. (1999). “The stress process revisited,” in *Handbook of the Sociology of Mental Health*, eds Aneshensel C. S. Phelan J. C. (Boston, MA: Springer), 395–415.

[B74] Pearlin L. I. Menaghan E. G. Lieberman M. A. Mullan J. T. (1981). The stress process. *J. Health Soc. Behav.* 22 337–356. 10.2307/2136676 7320473

[B75] Quassoli F. Dimitriadis I. (2019). “Here, There, in between, beyond”: identity negotiation and sense of belonging among Southern Europeans in the UK and Germany. *Soc. Incl. Soc. Incl.* 7:341. 10.17645/si.v7i4.2386

[B76] Russ T. C. Stamatakis E. Hamer M. Starr J. M. Kivimäki M. Batty G. D. (2012). Association between psychological distress and mortality: individual participant pooled analysis of 10 prospective cohort studies. *BMJ.* 345:e4933. 10.1136/bmj.e4933 22849956 PMC3409083

[B77] Ryan R. M. Sheldon K. M. Kasser T. Deci E. L. (1996). “All goals are not created equal: an organismic perspective on the nature of goals and their regulation,” in *The Psychology of Action: Linking Cognition and Motivation to Behavior*, eds Gollwitzer P. M. Bargh J. A. (New York, NY: The Guilford Press), 7–26.

[B78] Sapmaz F. Doğan T. Sapmaz S. Temizel S. Tel F. D. (2012). Examining predictive role of psychological need satisfaction on happiness in terms of self-determination theory. *Procedia Soc. Behav. Sci.* 55 861–868. 10.1016/j.sbspro.2012.09.573

[B79] Scheim A. I. Perez-Brumer A. G. Bauer G. R. (2020). Gender-concordant identity documents and mental health among transgender adults in the USA: a cross-sectional study. *Lancet Public Health* 5 e196–e203. 10.1016/S2468-2667(20)30032-3 32192577 PMC9912749

[B80] Scott D. Paterson J. L. Happell B. (2014). Poor sleep quality in Australian adults with comorbid psychological distress and physical illness. *Behav. Sleep Med.* 12 331–341. 10.1080/15402002.2013.819469 24180418

[B81] Sheldon K. M. Bettencourt B. A. (2002). Psychological need-satisfaction and subjective well-being within social groups. *Br. J. Soc. Psychol.* 41(Pt. 1) 25–38. 10.1348/014466602165036 11970772

[B82] Sina (2013). *The Ranking of New Cities in 2013 Has Been Released, and Two Cities of Liaoning Become New First-Tier Cities.* Available online at: http://ln.sina.com.cn/news/m/2013-12-13/080671645.html (accessed March 4 2021).

[B83] South E. C. Hohl B. C. Kondo M. C. MacDonald J. M. Branas C. C. (2018). Effect of greening vacant land on mental health of community-dwelling adults: a cluster randomized trial. *JAMA Network Open* 1:e180298. 10.1001/jamanetworkopen 30646029 PMC6324526

[B84] Spinosa J. Christiansen P. Dickson J. M. Lorenzetti V. Hardman C. A. (2019). From socioeconomic disadvantage to obesity: the mediating role of psychological distress and emotional eating. *Obesity* 27 559–564. 10.1002/oby.22402 30821100 PMC6593860

[B85] Steiger J. H. (1990). Structural Model evaluation and modification: an interval estimation approach. *Multivariate. Behav. Res.* 25 173–180. 10.1207/s15327906mbr2502_4 26794479

[B86] Steptoe A. Deaton A. Stone A. A. (2015). Subjective wellbeing, health, and ageing. *Lancet* 385 640–648. 10.1016/S0140-6736(13)61489-0 25468152 PMC4339610

[B87] Straiton M. L. Aambo A. K. Johansen R. (2019). Perceived discrimination, health and mental health among immigrants in Norway: the role of moderating factors. *BMC Publ. Health* 19:325. 10.1186/s12889-019-6649-9 30894173 PMC6425660

[B88] Sun J. Lyu S. J. (2020). Social participation and urban-rural disparity in mental health among older adults in China. *J. Affect. Disord.* 274 399–404. 10.1016/j.jad.2020.05.091 32663969

[B89] Switek M. (2016). Internal migration and life satisfaction: well-being paths of young adult migrants. *Soc. Indic. Res.* 125 191–241. 10.1007/s11205-014-0829-x

[B90] Tan J. J. X. Kraus M. W. Carpenter N. C. Adler N. E. (2020). The association between objective and subjective socioeconomic status and subjective well-being: a meta-analytic review. *Psychol. Bull.* 146 970–1020. 10.1037/bul0000258 33090862

[B91] Tesser A. Campbell J. Mickler S. (1983). The role of social pressure, attention to the stimulus, and self-doubt in conformity. *Eur. J. Soc. Psychol.* 13 217–233. 10.1002/ejsp.2420130303

[B92] Tian F. F. Qian Y. Qian Z. C. (2018). Hukou locality and intermarriages in two chinese cities: Shanghai and Shenzhen. *Res. Soc. Stratif. Mobil.* 56 12–20. 10.1016/j.rssm.2018.06.001 PMC8290908 34290467

[B93] Uphoff E. P. Pickett K. E. Cabieses B. Small N. Wright J. (2013). A systematic review of the relationships between social capital and socioeconomic inequalities in health: a contribution to understanding the psychosocial pathway of health inequalities. *Int. J. Equity Health* 12:54. 10.1186/1475-9276-12-54 23870068 PMC3726325

[B94] Viner R. M. Gireesh A. Stiglic N. Hudson L. D. Goddings A.-L. Ward J. L. (2019). Roles of cyberbullying, sleep, and physical activity in mediating the effects of social media use on mental health and wellbeing among young people in England: a secondary analysis of longitudinal data. *Lancet Child Adolesc. Health.* 3 685–696. 10.1016/S2352-4642(19)30186-5 31420213

[B95] Wang D. Y. Hu M. M. Xu Q. F. (2017). Testing the factorial invariance of the satisfaction with life scale across chinese adolescents. *Soc. Behav. Pers.* 45 505–516. 10.2224/sbp.6222

[B96] Wang K. T. Yuen M. Slaney R. B. (2009). Perfectionism, depression, loneliness, and life satisfaction a study of high school students in Hong Kong. *Couns. Psychol.* 37 249–274. 10.1177/0011000008315975

[B97] Wang R. Feng Z. Liu Y. Lu Y. (2020). Relationship between neighbourhood social participation and depression among older adults: a longitudinal study in China. *Health Soc. Care Commun.* 28 247–259. 10.1111/hsc.12859 31595604

[B98] Wang W. Fan C. C. (2012). Migrant workers’ integration in urban China: experiences in employment, social adaptation, and self-identity. *Eur. Geogr. Econ.* 53 731–749. 10.2747/1539-7216.53.6.731

[B99] Wei L. Gao F. F. (2017). Social media, social integration and subjective well-being among new urban migrants in China. *Telemat. Inform.* 34 786–796. 10.1016/j.tele.2016.05.017

[B100] Wong D. F. He X. Leung G. Lau Y. Chang Y. (2008). Mental health of migrant workers in China: prevalence and correlates. *Soc. Psychiatry Psychiatr. Epidemiol.* 43 483–489. 10.1007/s00127-008-0341-1 18398559

[B101] World Health Organization (WHO) (2016). *Suicide (2016). [Online].* Available online at: https://www.who.int/en/news-room/fact-sheets/detail/suicide (accessed July 5 2020).

[B102] Yang F.-J. (2020). The “How” question of the healthy immigrant paradox: understanding psychosocial resources and demands as pathways linking migration to mental health risks. *Soc. Ment. Health* 11 69–89. 10.1177/2156869320913090

[B103] Ye S. Q. (2007). Validation of the temporal satisfaction with life scale in a sample of Chinese university students. *Soc. Indic. Res.* 80 617–628. 10.1007/s11205-006-0010-2

[B104] Yıldız M. Duy B. (2014). Adaptation of the short-form of the UCLA loneliness scale (ULS-8) to Turkish for the adolescents. *Dusunen Adam.* 27 194–203. 10.5350/DAJPN2014270302

[B105] You J. Zhu Y. Liu S. Q. Wang C. Wang P. G. Du H. F. (2019). Socioeconomic disparities in psychological health: testing the reserve capacity model in a population-based sample of Chinese migrants. *J. Health Psychol* 1359105319882763. 10.1177/1359105319882763 [Epub ahead of print]. 31621415

[B106] Yuan H. (2016). Structural social capital, household income and life satisfaction: the evidence from Beijing, Shanghai and Guangdong-province, China. *J. Happiness Stud.* 17 569–586. 10.1007/s10902-015-9622-z

[B107] Zechmann A. Paul K. I. (2019). Why do individuals suffer during unemployment? analyzing the role of deprived psychological needs in a six-wave longitudinal Study. *J. Occup. Health Psychol.* 24 641–661. 10.1037/ocp0000154 30945924

[B108] Zevallos Z. (2008). ‘You have to be Anglo and not look like me’: identity and belonging among young women of Turkish and Latin American backgrounds in Melbourne, Australia. Australian Aust. *Geographer. Geogr.* 39 21–43. 10.1080/00049180701877410

[B109] Zhang J. T. Li X. M. Fang X. Y. Xiong Q. (2009). Discrimination experience and quality of life among rural-to-urban migrants in China: the mediation effect of expectation-reality discrepancy. *Qual. Life Res.* 18 291–300. 10.1007/s11136-009-9454-6 19225905

[B110] Zheng Y. T. Ji Y. Chang C. Liverani M. (2020). The evolution of health policy in China and internal migrants: continuity, change, and current implementation challenges. *Asia Pac. Policy Stud.* 7 81–94. 10.1002/app5.294

