# Supplementary material for: Influence of Subjective/Objective Status and Possible Pathways of Young Migrants’ Life Satisfaction and Psychological Distress in China
Source: Front Psychol. 2021 May 26;12:612317. doi: 10.3389/fpsyg.2021.612317 (PMC8187866; doi:10.3389/fpsyg.2021.612317)
Supplement: Supplementary file 1 [file Table_1.DOCX]

**Supplementary Table 1 |** Correlation Matrix of the Latent Exogenous Variables (Subjective and Objective Indicators of Status) according to the final SEM estimation by using LISREL 8.80.

| **Latent exogenous variables** | **S_SES** |  | **S_SA** |  | **S_PI** |  | **S_SE** |  | **O_SES** |  | **O_HI** |
| --- | --- | --- | --- | --- | --- | --- | --- | --- | --- | --- | --- |
| S_SES | 1.00 | -- |  |  |  |  |  |  |  |  |  |
| S_SA | 0.03 | ^**^ | 1.00 | -- |  |  |  |  |  |  |  |
| S_PI | 0.09 | ^***^ | 0.07 | ^***^ | 1.00 | -- |  |  |  |  |  |
| S_SE | -0.08 | ^***^ | -0.05 | ^**^ | -0.43 | ^***^ | 1.00 | -- |  |  |  |
| O_SES | 0.05 | ^*^ | 0.41 | ^*^ | 0.10 | ^*^ | -0.05 |  | 1.00 | -- |  |
| O_HI | 0.04 |  | 0.30 |  | 0.11 |  | -0.11 |  | 0.70 |  | 1.00 |

*S_SES, Subjective Socioeconomic Status; S_SA, Social Adaptation; S_PI, Psychological Integration; S_SE, Social Exclusion; O_SES, Objective Socioeconomic Status; O_HI, Health Insurance.*

**p <* *0.05 (two-tailed significance tests);*

***p <* *0.01 (two-tailed significance tests);*

****p < 0.001(two-tailed significance tests).*
